# Simultaneous quantitative detection of multiple low-frequency variants by high-dynamic-range capillary electrophoresis

**DOI:** 10.1016/j.omtn.2026.103042

**Published:** 2026-07-30

**Authors:** Yoshihiko Hagiwara, Nobue Tamamura, Noriyuki Sumida, Yusuke Ono, Kenji Takahashi, Yanpeng Sun, Kazuya Koyama, Yu Ohtaki, Tetsuhiro Okada, Hidemasa Kawabata, Chiho Maeda, Miyuki Mori, Chiemi Matsumoto, Mayumi Suzuki, Shin-ichi Chiba, Mishie Tanino, Kenzui Taniue, Hirokazu Kato, Motohiro Yamazaki, Yusuke Mizukami

**Affiliations:** 1 Hitachi High-Tech Corporation, Tokyo 105-6409, Japan; 2 Department of Advanced Genomic Community Healthcare, Asahikawa Medical University, Asahikawa, Hokkaido 078-8510, Japan; 3 Division of Gastroenterology, Department of Medicine, Asahikawa Medical University, Asahikawa, Hokkaido 078-8510, Japan; 4 Institute of Biomedical Research, Sapporo-Higashi Tokushukai Hospital, Sapporo, Hokkaido 065-0033, Japan; 5 Center for Intractable Diseases and ImmunoGenomics, National Institute of Biomedical Innovation Health and Nutrition, Ibaraki, Osaka 567-0085, Japan; 6 Center for Advanced Research and Education, Asahikawa Medical University, Asahikawa, Hokkaido 078-8510, Japan; 7 Department of Diagnostic Pathology, Asahikawa Medical University Hospital, Asahikawa, Hokkaido 078-8510, Japan; 8 Isotope Science Center, The University of Tokyo, Tokyo 113-0032, Japan

**Keywords:** MT: clinical applications, molecular diagnostics, genetic analysis, HiDy, capillary electrophoresis, multiplex shifted termination assay, dynamic range, quantitative analysis, cancer, pancreatic disease

## Abstract

Sensitive and quantitative detection of low-frequency variants across multiple loci is critical for nucleic acid-based diagnostics, yet clinical implementation requires a balance among sensitivity, multiplexing capacity, cost, and operational simplicity. We previously developed a high-dynamic-range capillary electrophoresis system capable of detecting variants at allele frequencies below 1%; however, its application was limited to single-locus analysis. Here, we expanded this platform to multiplex detection by incorporating mobility-shift strategies into the assay design. This approach enabled simultaneous analysis of 15 hotspot variants across three clinically relevant loci: *KRAS* codons 12 and 13 and *GNAS* codon 201. Validation using synthetic oligonucleotides, formalin-fixed paraffin-embedded tissue, and liquid specimens demonstrated high quantitative accuracy over clinically relevant variant allele frequency ranges, with measured values closely matching expected values (R^2^ > 0.97). The assay showed high concordance with targeted amplicon sequencing and digital polymerase chain reaction for all variants at variant allele frequencies ≥1%, while also detecting selected variants below this threshold. Collectively, these results establish a multiplexed high-dynamic-range capillary electrophoresis assay for simultaneous, quantitative detection of low-frequency variants, offering a scalable and cost-effective approach for disease-focused gene panels in clinical laboratory settings.

## Graphical abstract

**Figure:**
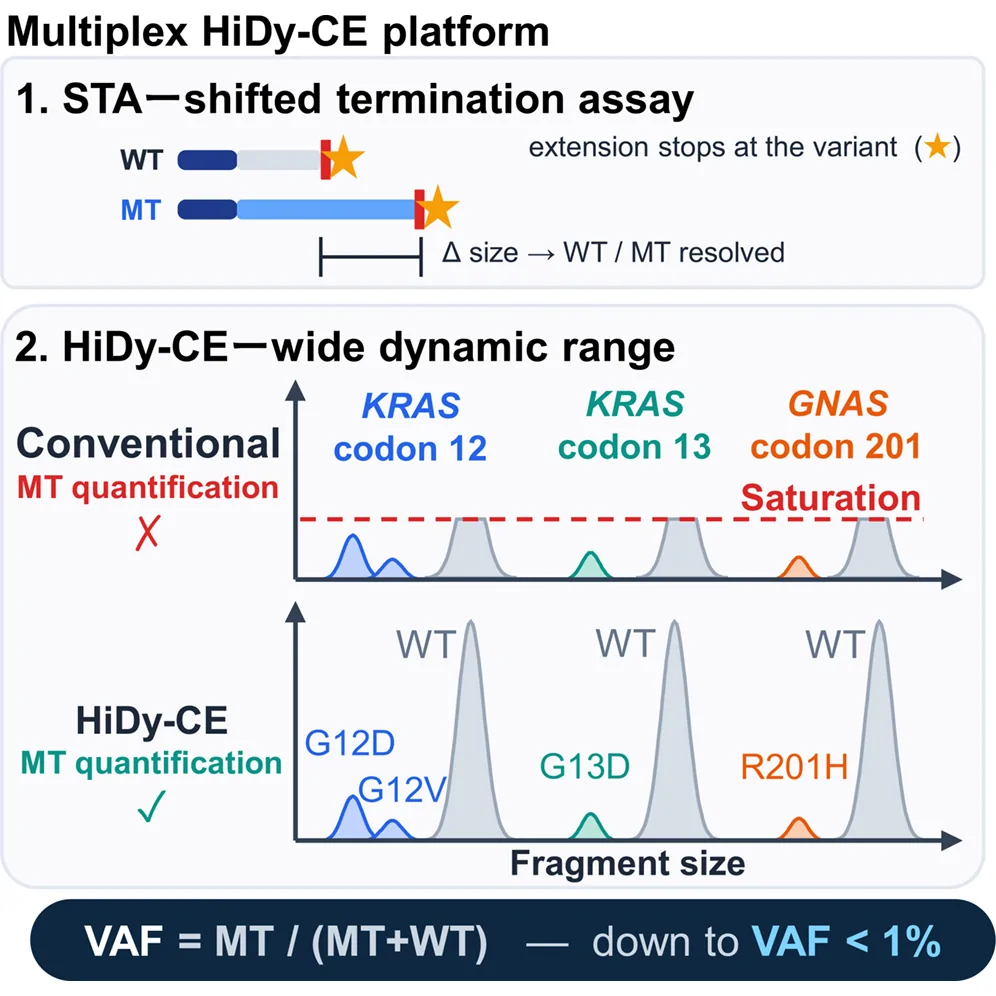

## Introduction

Simultaneous detection of low-frequency somatic variants across multiple genomic loci remains an unmet need in targeted nucleic acid-based diagnostics.^1^ Accurate identification of driver mutations present at low variant allele frequencies (VAFs) requires assay platforms that combine high analytical sensitivity with robust quantitative performance.^2^ Despite technological advances, routine clinical testing still faces practical constraints in multiplexing capacity, sample input requirements, cost, and analytical throughput.^3^^,^^4^^,^^5^ There is therefore a need for scalable and operationally simple analytical systems that enable reliable quantification of low-abundance variants in clinical laboratory settings.^6^

Capillary electrophoresis (CE) offers high-resolution nucleic acid separation and is inherently compatible with multiplex assays.^7^^,^^8^ However, conventional CE sequencers suffer from fluorescence signal saturation by abundant wild-type (WT) DNA, resulting in restricted dynamic range that compromises quantification of low-frequency variants.^9^ To address this limitation, we previously developed high-dynamic-range capillary electrophoresis (HiDy-CE),^10^ which enables sensitive detection of minute mutant alleles even in the presence of strong WT signals. While this approach demonstrated analytical utility in clinical biopsy specimens, its initial application was limited to single-locus analysis. In multiplex CE-based analysis, multiple high-intensity WT peaks are generated within a single run. The broad dynamic range of HiDy-CE, however, provides a technical basis for discrimination and quantification of multiple low-frequency variants without signal saturation, supporting its application in targeted molecular diagnostics and nucleic acid-driven precision medicine.^7^^,^^8^^,^^10^

Pancreatic neoplasms provide a suitable model for evaluating sensitive multiplex mutation detection because early driver mutations are well characterized and often occur at low VAFs.^11^^,^^12^ Activating *KRAS* mutations at codons 12 and 13 are among the earliest and most prevalent alterations in pancreatic ductal adenocarcinoma (PDA) and related precursor lesions,^11^^,^^13^^,^^14^^,^^15^ whereas activating *GNAS* mutations at codon 201 are characteristic of intraductal papillary mucinous neoplasms (IPMNs).^13^^,^^16^^,^^17^ These mutations serve as well-defined molecular targets for analytical validation of sensitive mutation assays.^13^

In this study, we established a multiplex detection and quantification system targeting 15 hotspot variants across *KRAS* codons 12 and 13 and *GNAS* codon 201 by integrating a shifted termination assay (STA) with the HiDy-CE platform. We evaluated the analytical performance of this system using surgically resected tissues and liquid specimens from PDA and IPMN.

## Results

### Establishment of the multiplex STA-HiDy-CE assay

We established a multiplex STA coupled with HiDy-CE for simultaneous detection of 15 variants across 3 loci, including 6 *KRAS* codon 12 variants, 6 *KRAS* codon 13 variants, and 3 *GNAS* codon 201 variants (Table S1).

**Figure 1:**
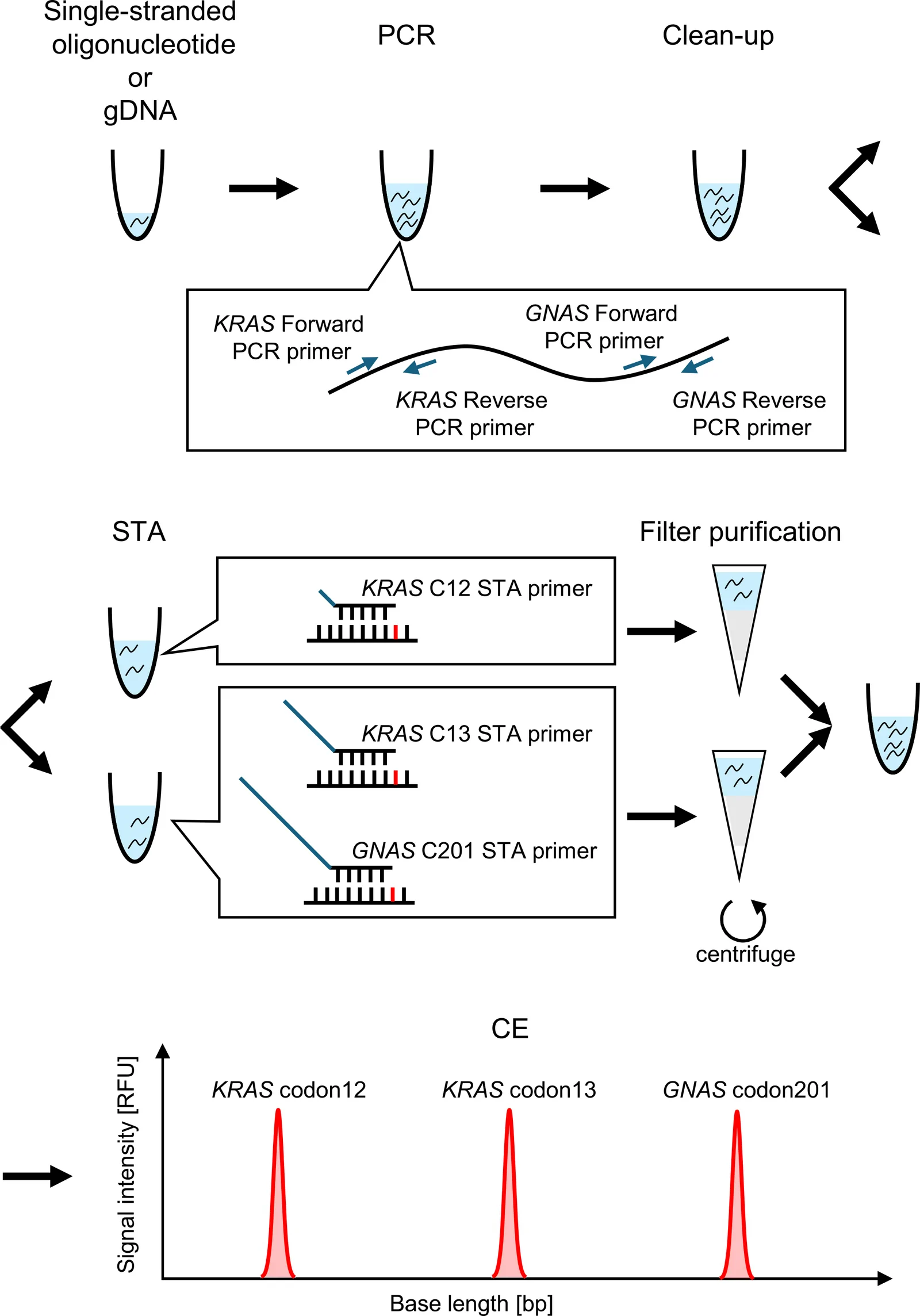
Schematic overview of the multiplex STA-HiDy-CE system K*RAS* and *GNAS* targets were co-amplified, purified, and subjected to shifted termination assay (STA). STA reactions were performed in 2 tubes, followed by filter purification of each reaction product. In a single capillary electrophoresis run, *KRAS* codons 12 and 13 and *GNAS* codon 201 were resolved at distinct fragment sizes according to STA primer design.

**Figure 2:**
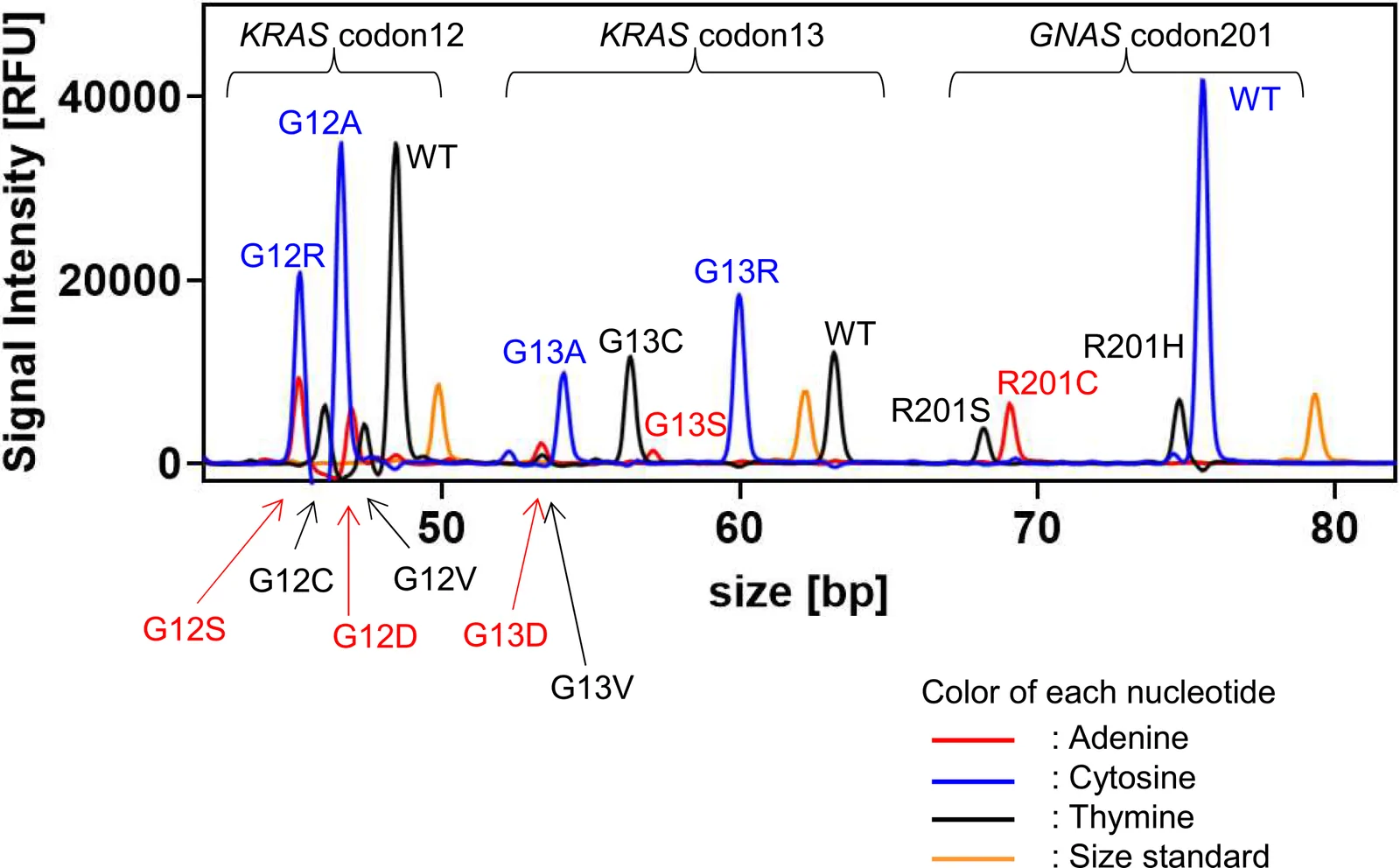
Establishment of the multiplex STA-HiDy-CE system Representative electropherogram of synthetic single-stranded oligonucleotide mixture containing all 15 detectable variants. *KRAS* codons 12 and 13, and *GNAS* codon 201 allele were resolved at different fragment sizes owing to STA primer design. Peak colors indicate the base type detected.

To enable multiplex detection, polymerase chain reaction (PCR) primers were designed to co-amplify *KRAS* and *GNAS* targets in a single reaction, and STA primers were designed to generate electrophoretically distinguishable products corresponding to *KRAS* codons 12 and 13 and *GNAS* codon 201 (Figure 1; Table S2). When synthetic single-stranded DNA (ssDNA) representing all 15 variants and their corresponding WT alleles were mixed such that each variant accounted for 8% of the alleles at each locus, all 15 variants were resolved as distinct, non-overlapping peaks on a single electropherogram, demonstrating successful multiplex detection by the STA-HiDy-CE system (Figure 2).

During assay development, we observed reproducible cross-signal artifacts in which certain variants generated low-level peaks at positions corresponding to other alleles. In particular, a G12D-like peak was observed in samples containing only G12V, and an R201S-like peak was observed in samples containing only R201C; the magnitude of these signals increased in proportion to the abundance of the source variant (Figure S1). To address this issue, we implemented a matrix-based noise subtraction method based on empirically derived noise patterns (Table S3; materials and methods).

### Analytical performance of multiplex STA-HiDy-CE

To evaluate analytical performance, synthetic oligonucleotides containing all 15 detectable variants were serially diluted such that all variants were present at equal proportions across an input VAF range from 0% to 8%. Raw peak heights differed among variants despite equal input proportions, indicating systematic peak-height variation associated with fluorophore characteristics, sequence composition, and fragment size. To correct this effect, we derived variant-specific correction coefficient relative to WT signal intensity and incorporated these coefficients into the VAF calculation algorithm (Table S4; materials and methods).

**Figure 3:**
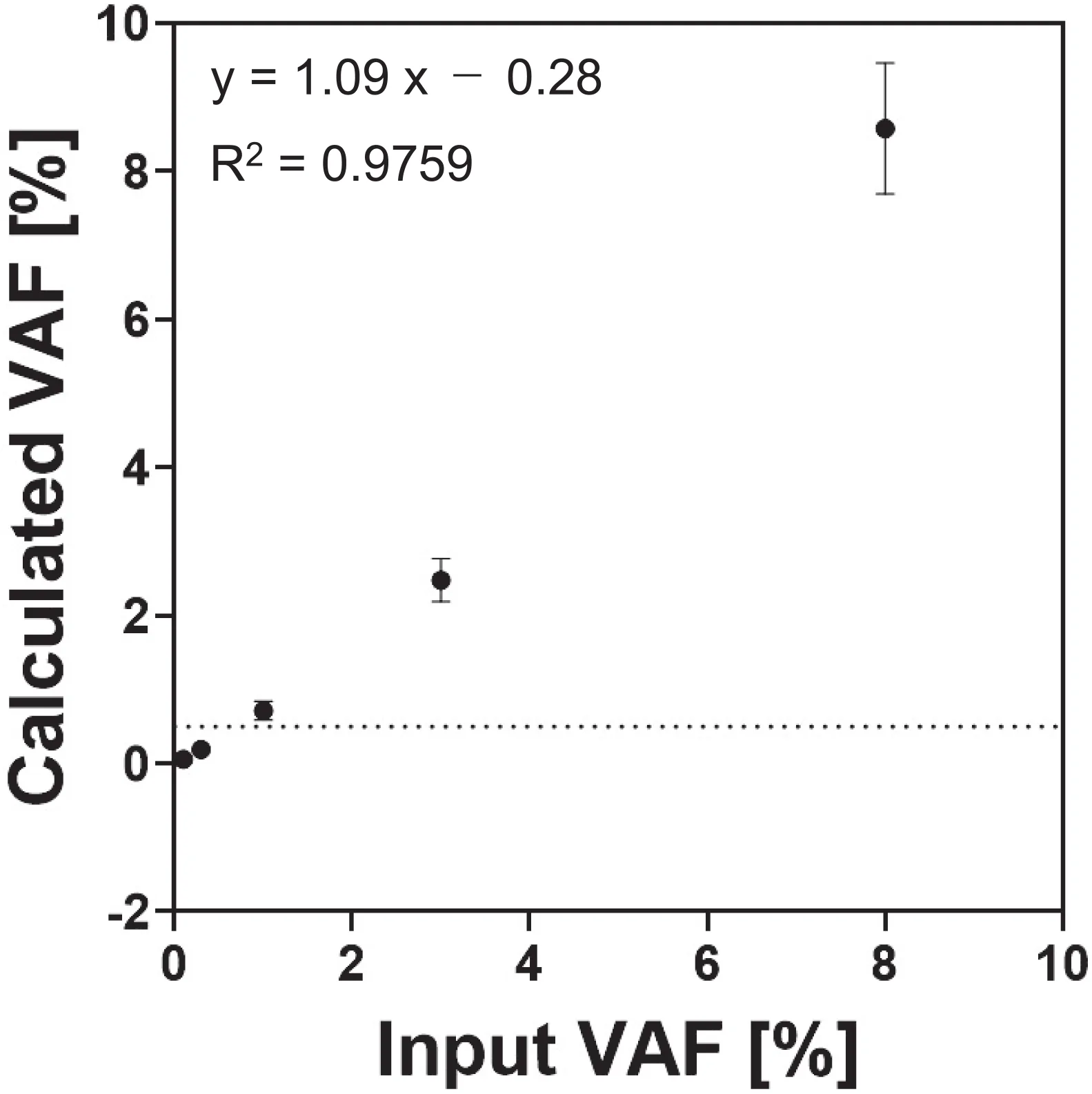
Calibration curve on multiplex STA-HiDy-CE using control DNA Calibration curves generated from dilution series experiment. Six replicates were analyzed for each variant allele frequency (VAF). The *x* axis indicates the input VAF, the *y* axis indicates the calculated VAF according to Equations 3 or 4. Dots, error bars, and dashed line represent mean values, standard deviations, and the mutation-calling threshold, respectively.

**Figure 4:**
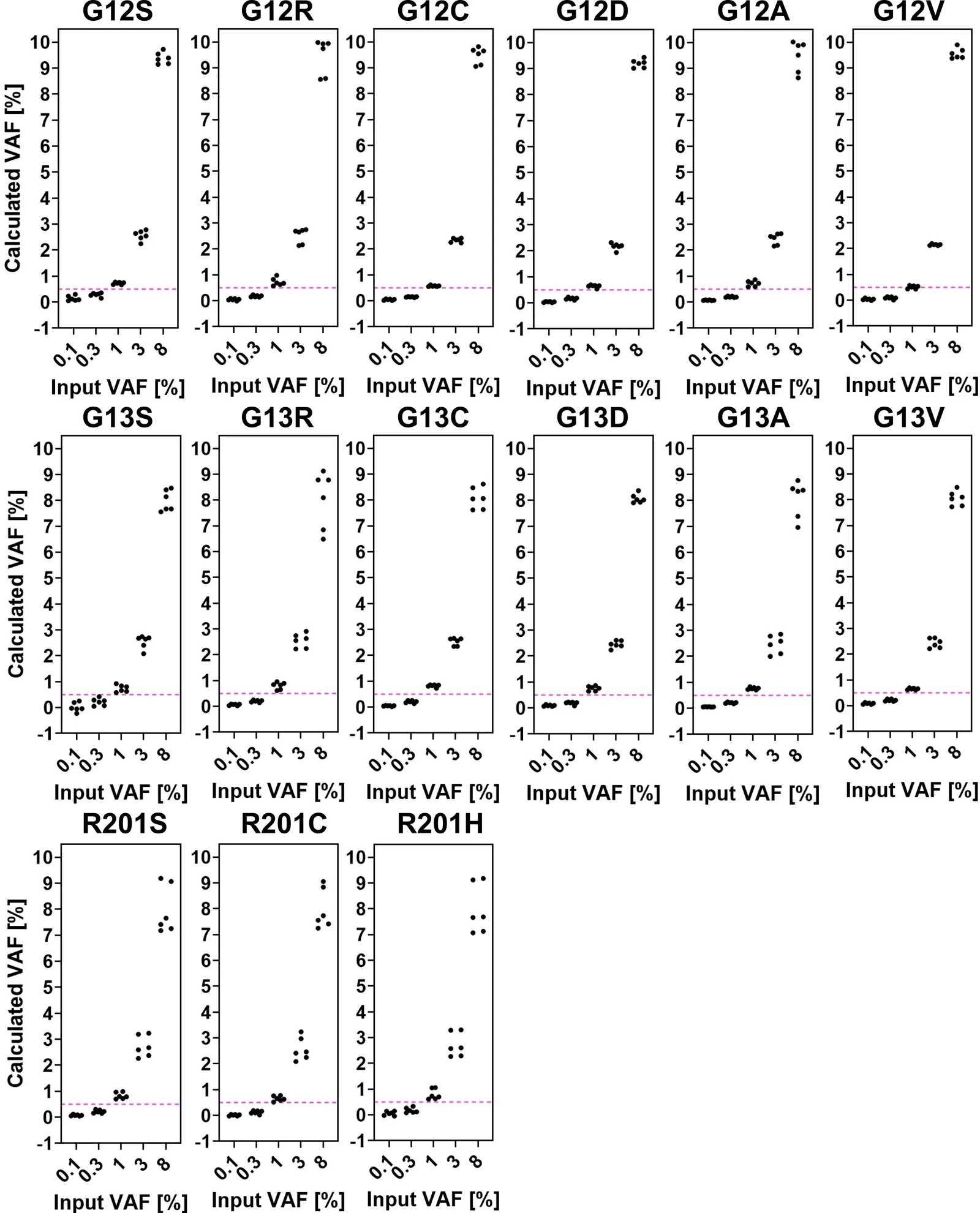
Calculated VAFs for each variant using control sample Dot plots of calculated variant allele frequencies (VAFs) for each variant using synthetic single-stranded oligonucleotides. The 0.5% threshold for mutation calling is indicated by a magenta dashed line.

After correction, measured VAFs showed strong agreement with input VAFs across the dilution series, demonstrating robust quantitative performance of the multiplex STA-HiDy-CE assay (Figure 3). The coefficient of determination was 0.9759, and the slope and *y*-intercept of the regression line were approximately 1 and 0, respectively, indicating a high degree of linearity (Figure 3). At input VAFs of 0.1% and 0.3%, no variants exceeded the 0.5% mutation-calling threshold. In contrast, at input VAFs of 1.0% or greater, 99.26% of measurements exceeded the threshold (Figure 4; Table 1). Using the same dilution series data, coefficient of variation (CV) and within-run CVs were calculated. CVs were <20% at input VAFs of 1% or higher, demonstrating high and consistent precision across measurement days (Table 1). Accuracy was <20% at input VAF of 3% or higher (Table 1). Based on predefined criteria requiring both accuracy and precision to be <20%, the limit of quantification (LOQ) was determined to be 3% VAF, as accuracy criteria were not met below this threshold.

**Table 1:** Analytical performance of dilution series experiment

Input VAF (%)	Mean value of calculated VAF (%)	Accuracy deviation (%)	Sensitivity (%)	Within-run CV (%)Day 1	Within-run CV (%)Day 2	Total CV (%)
0.1	0.06	37.88	0	112.80	93.21	103.20
0.3	0.19	36.07	0	36.47	38.80	37.67
1	0.72	28.38	97.78	17.14	18.91	18.11
3	2.48	17.32	100	12.41	10.98	11.82
8	8.58	7.28	100	10.97	9.63	10.34

### Performance in FFPE tissue samples

We next evaluated the assay performance in clinical formalin-fixed, paraffin-embedded (FFPE) tissue specimens. Forty samples from patients with pancreaticoduodenal tumors were analyzed, including specimens harboring *KRAS* and/or *GNAS* mutations and mutation-negative control samples (Table 2). For each assay, 10 ng of genomic DNA (gDNA )were used as input, and all samples were analyzed in duplicate.

**Table 2:** Mutation detection capability on multiplex STA-HiDy-CE system using tissue samples

Sample	Pathologic diagnosis	DNA concentration by Qubit [ng/μL]	DNA yield by Qubit [ng]	Multiplex STA-HiDy-CE				Targeted amplicon sequencing			
				*KRAS* mutation	VAF [%]	*GNAS* mutation	VAF [%]	*KRAS* mutation	VAF [%]	*GNAS* mutation	VAF [%]
1	HG IPMN	18.9	94.5	G12D	4.9	ND	–	G12D	7.4	ND	–

2	LG PanIN	40	200	G12R	7.0	R201C	8.7	G12R	8.3	R201C	10.1

–	–	–	–	G12S	0.5	–	–	–	–	–	–

3	LG IPMN	4.85	53.35	ND	–	R201H	17.8	ND	–	R201H	18.1

4	LG IPMN	46.4	232	G12V	2.8	R201H	2.6	G12V	3.1	R201H	3

–	–	–	–	G12R	0.8	–	–	∗	–	–	–

5	LG IPMN	42.2	211	G12D	41.4	R201H	40.8	G12D	44.4	R201H	43.5

6	LG IPMN	8.78	52.68	G12C	4.1	ND	–	G12C	3.8	ND	–

	–	–	–	G12D	1.2	–	–	G12D	1	–	–

7	LG IPMN	8.89	53.34	G12D	19.2	R201S	18.2	G12D	21.8	R201S	23.3

–	–	–	–	–	–	–	–	–	–	–	–

8	LG IPMN	26.3	131.5	G12V	21.5	R201H	18.7	G12V	19.7	R201H	19.1

–	–	–	–	G12A	1.1	–	–	G12A	2.2	–	–

–	–	–	–	G12S	0.7	–	–	–	–	–	–

9	carcinoma	23	115	G12R	8.1	ND	–	G12R	10.2	ND	–

10	LG IPMN	27.4	137	ND	–	R201C	17.4	ND	–	R201C	17.4

11	HG PanIN	4.18	50.16	G12V	5.5	ND	–	G12V	5.1	ND	–

12	HG IPMN	25.4	76.2	G12V	41.9	R201C	47.6	G12V	42.5	R201C	42
13	HG IPMN	4.26	51.12	G12R	6.8	ND	–	G12R	4.1	ND	–
–	–	–	–	G12V	1.2	–	–	∗	–	–	–
–	–	–	–	G12S	0.8	–	–	–	–	–	–
14	HG IPMN	56	280	G12D	56.3	ND	–	G12D	61.8	ND	–
–	–	–	–	G12S	0.5	–	–	–	–	–	–
15	LG IPMN	34.6	173	G12V	4.8	R201H	3.8	G12V	4.8	R201H	4.9
16	LG IPMN	11.4	57	G12R	11.3	R201C	14.2	G12R	12.6	R201C	14.5
17	LG IPMN	18.7	93.5	G12V	30.8	R201H	30.7	G12V	31.8	R201H	33.2
18	LG IPMN	10.4	52	G12D	23.6	R201H	23.6	G12D	27.16	R201H	27.35
19	carcinoma	6.66	33.3	G13D	28	ND	–	G13D	26.01	ND	–
20	LG IPMN	56	112	G13D	9.7	R201H	8.4	G13D	9.9	R201H	11
21	spleen	35	175	ND	–	ND	–	ND	–	ND	–
22	duodenum	15.9	79.5	ND	–	ND	–	ND	–	ND	–
23	duodenum	23.7	118.5	ND	–	ND	–	ND	–	ND	–
24	spleen	173	865	ND	–	ND	–	ND	–	ND	–
25	spleen	52	260	G12S	0.5	ND	–	ND	–	ND	–
26	duodenum	64.8	324	ND	–	ND	–	ND	–	ND	–
27	duodenum	87.8	439	ND	–	ND	–	ND	–	ND	–
28	spleen	104	520	ND	–	ND	–	ND	–	ND	–
29	spleen	112	560	ND	–	ND	–	ND	–	ND	–
30	duodenum	35	175	ND	–	ND	–	ND	–	ND	–
31	CP/AIP	3.34	60	ND	–	ND	–	ND	–	ND	–
32	CP/AIP	15.6	281	ND	–	ND	–	ND	–	ND	–
33	CP/AIP	17.6	317	ND	–	ND	–	ND	–	ND	–
34	CP/AIP	11.4	205	ND	–	ND	–	ND	–	ND	–
35	CP/AIP	5.03	91	G13V	0.6	ND	–	ND	–	ND	–
36	CP/AIP	4.25	77	ND	–	ND	–	ND	–	ND	–
37	CP/AIP	56	1008	ND	–	ND	–	ND	–	ND	–
38	CP/AIP	33.9	610	ND	–	ND	–	ND	–	ND	–
39	CP/AIP	27.7	499	ND	–	ND	–	ND	–	ND	–
40	CP/AIP	3.1	56	ND	–	ND	–	ND	–	ND	–

**Figure 5:**
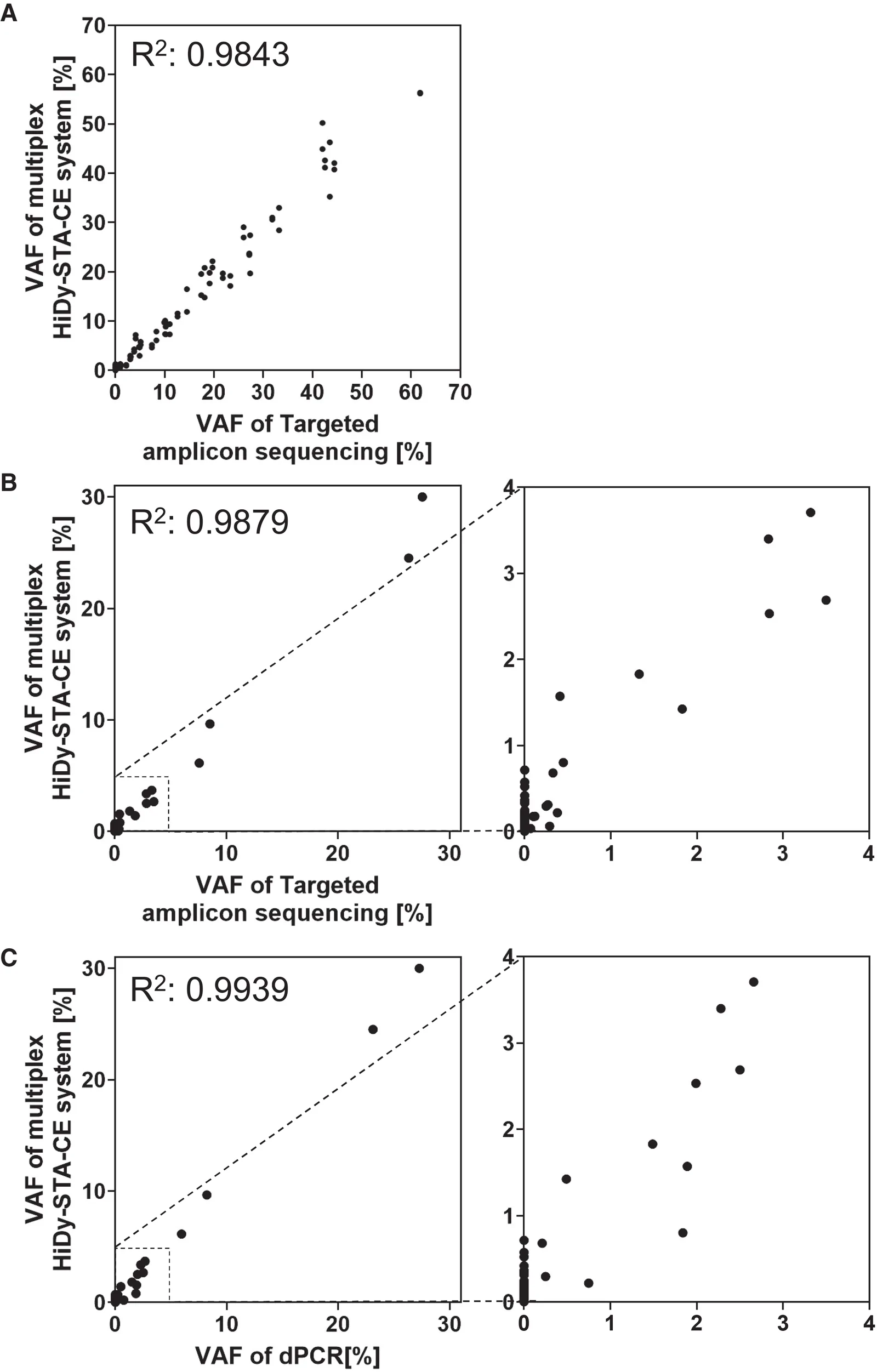
Correlation of VAFs between multiplex STA-HiDy-CE and reference methods Correlation of variant allele frequencies (VAFs) obtained by the multiplex shifted termination assay-high dynamic range capillary electrophoresis system (STA-HiDy-CE) and reference methods. (A) Comparison with targeted amplicon sequencing using FFPE specimens. (B) Comparison with targeted amplicon sequencing using liquid specimens. (C) Comparison with digital PCR using liquid specimens. All calculated VAF values, including those below the mutation-calling threshold, are plotted.

All mutations identified by targeted amplicon sequencing were also detected by the multiplex STA-HiDy-CE assay. With targeted amplicon sequencing as the reference method, false-positive calls were observed, yielding a specificity of 98.59% (Tables S5 and S6). Of the 8 observed false-positive calls, 2 were not identified by targeted amplicon sequencing; however, corresponding sequencing reads were present on visual inspection using Integrative Genomics Viewer (patient #4, G12R, 1.5%, 70/4721 reads; patient #13, G12V, 2.3%, 43/1851 reads). These findings suggest that the multiplex STA-HiDy-CE system may detect variants not called by targeted amplicon sequencing. Quantitative comparison showed strong agreement between VAFs obtained by multiplex STA-HiDy-CE and targeted amplicon sequencing (Figure 5A; *R*^2^ = 0.9843).

### Performance in liquid specimens

We next assessed assay performance in liquid specimens, including pancreatic juice and duodenal fluid (Table 3). Ten liquid specimens were analyzed by multiplex STA-HiDy-CE, targeted amplicon sequencing, and digital PCR. For multiplex STA-HiDy-CE, 10 ng of input gDNA was used for each assay.

**Table 3:** Mutation detection capability on multiplex STA-HiDy-CE system using liquid samples

Sample	Sample type	DNA concentration by Qubit [ng/μL]	DNA yield by Qubit [ng]	Multiplex STA-HiDy-CE				Targeted amplicon sequencing				Digital PCR			
				*KRAS* mutation	VAF [%]	*GNAS* mutation	VAF [%]	*KRAS* mutation	VAF [%]	*GNAS* mutation	VAF [%]	*KRAS* mutation	VAF [%]	*GNAS* mutation	VAF [%]
41	duodenal fluid	55	275	G12V	1.4	ND	–	G12V	1.83	ND	–	G12V	0.49	ND	–
42	pancreatic juice	3.57	35.7	G12V	30	ND	–	G12V	27.55	ND	–	G12V	27.26	ND	–
–	–	–	–	G12D	0.8	–	–	G12D	0.45	ND	–	G12D	1.84	ND	–
–	–	–	–	–	–	–	–	G12R	0.38	ND	–	G12R	0.75	ND	–
–	–	–	–	–	–	–	–	G12C	0.27	ND	–	–	–	ND	–
43	duodenal fluid	42.8	214	ND	–	ND	–	ND	–	ND	–	ND	–	ND	–
44	duodenal fluid	12.3	61.5	ND	–	ND	–	G12V	0.12	R201H	0.06	–	–	ND	–
–	–	–	–	–	–	–	–	–	–	–	–	G12D	0.11	–	–
45	pancreatic juice	47.4	237	G12D	1.8	ND	–	G12D	1.33	ND	–	G12D	1.49	ND	–
–	–	–	–	–	–	–	–	G12R	0.29	ND	–	–	–	ND	–
46	duodenal fluid	10.7	53.5	ND	–	ND	–	G12V	0.1	ND	–	–	–	ND	–
–	–	–	–	–	–	–	–	G12D	0.06	ND	–	G12D	0.17	ND	–
–	–	–	–	–	–	–	–	G12R	0.06	ND	–	–	–	ND	–
47	duodenal fluid	32.1	160.5	G12V	2.7	R201C	3.7	G12V	3.5	R201C	3.32	G12V	2.5	R201C	2.66
48	duodenal fluid	5.18	51.8	G12V	2.5	R201C	3.4	G12V	2.84	R201C	2.83	G12V	1.99	R201C	2.28
–	–	–	–	–	–	R201H	0.7	G12C	0.07	R201H	0.33	–	–	R201H	0.21
49	pancreatic juice	3.27	32.7	G12V	9.7	R201H	24.5	G12V	8.52	R201H	26.34	G12V	8.21	R201H	23.12
–	–	–	–	G12D	1.6	–	–	G12D	0.41	R201C	0.25	G12D	1.89	R201C	0.25
50	duodenal fluid	5.35	53.5	G12V	6.1	ND	–	G12V	7.57	ND	–	G12V	5.94	ND	–

Hagiwara, Tamamura, and colleagues developed a multiplex high-dynamic-range capillary electrophoresis assay for simultaneous detection of low-frequency variants across multiple loci. By incorporating mobility-shift strategies, the assay achieves a practical balance among sensitivity, multiplexing capacity, cost, and operational simplicity, supporting implementation of disease-focused gene panels in clinical laboratory settings.

Among the mutations identified by digital PCR, all variants with VAFs of 1% or greater were detected by multiplex STA-HiDy-CE. Of 6 variants with VAFs below 1%, 2 were detected by the assay. Using digital PCR as the reference method, false-positive calls were also observed in liquid specimens, yielding a specificity of 97.74% (Tables S6 and S7). Quantitative comparison demonstrated strong agreement between VAFs by multiplex STA-HiDy-CE and those obtained by targeted amplicon sequencing and digital PCR (Figures 5B and 5C; *R*^2^ = 0.9879 and 0.9939, respectively).

Together, these findings indicate that the multiplex STA-HiDy-CE assay enables simultaneous detection and quantitative analysis of multiple variants in liquid clinical specimens, with consistent detection of variants at VAFs of 1% or greater and partial detection below this level.

## Discussion

In this study, we established a multiplex STA combined with HiDy-CE system that enables simultaneous detection and quantification of multiple low-frequency variants within a single electrophoretic run. By integrating multiplex STA chemistry with an expanded dynamic-range CE platform, this system addresses a key limitation of conventional CE-based assays, which have historically been restricted to single-locus analysis or have shown limited quantitative performance for low-allele frequency variants.

Analytical validation using FFPE tissue and liquid specimens demonstrated high concordance with established reference methods, including targeted amplicon sequencing and digital PCR. In this study, *KRAS* and *GNAS* mutations were used as representative analytical targets to evaluate multiplex performance in clinically relevant specimens. The ability to resolve and quantify multiple low-frequency variants across loci highlights the potential utility of this approach as a targeted and scalable molecular analysis platform for clinical laboratories.

Accurate quantification of VAFs becomes increasingly challenging when multiple variants coexist, particularly within the same genomic locus. In the multiplex STA-HiDy-CE system, electropherograms generated from samples containing all 15 detectable variants showed that raw peak-height ratios did not directly reflect the actual variant proportions (Figure 2). In addition, certain variants generated characteristic cross-signal artifacts, as shown in Figure S1. These discrepancies are likely attributable to systematic differences among variants, including fluorophore properties, sequence composition, and fragment size-dependent electrophoretic behavior. To address these issues, we introduced variant-specific correction coefficients to normalize peak-height bias and implemented a noise-cancellation algorithm to reduce cross-signal artifacts. Together, these approaches enabled improved quantification of multiple variants, including in samples containing a complex mixture of mutant alleles, as may occur in clinical tissue and liquid specimens. This analytical framework therefore provides a practical solution for multiplex quantitative mutation analysis in CE-based assays.

Previous CE-based studies have demonstrated sensitive detection of low-frequency variants at a single locus using shifted termination, single-base extension, or Sanger sequencing approaches.^10^^,^^18^ STA offers improved separation of WT and mutant alleles compared with conventional single-base extension methods, including SNaPShot, because it generates size-resolved products.^10^^,^^18^^,^^19^ In addition, detection of variants below 1% VAF has been reported using cell line-derived gDNA.^8^ However, to our knowledge, multiplex detection and quantification of low-frequency variants across multiple loci in clinical specimens has not previously been demonstrated using CE-based platforms. In the present study, we adapted the primer-modification strategy originally developed for single-base extension assay by appending non-hybridizing sequences to STA primers,^8^ thereby enabling controlled electrophoretic separation across loci. When combined with the expanded dynamic range of HiDy-CE, this strategy enabled simultaneous detection and quantification of multiple low-frequency variants within a single electrophoretic run.

In this proof-of-concept study, multiplex analysis was limited to 3 loci within a fragment size window of approximately 35 bp (45–80 bp). Because CE provides single-base resolution across several hundred base pairs,^20^ substantial expansion of multiplex capacity appears feasible. A simple estimation suggests that several dozen single-nucleotide variants could potentially be resolved within a single run, depending on primer design and size spacing. Further optimization of STA primer architecture and fluorophore combinations may therefore increase the number of targetable loci.

From an analytical perspective, the multiplex STA-HiDy-CE system occupies a complementary position among existing molecular testing platforms. Although comprehensive genomic profiling (CGP) provides broad mutational coverage, it is resource-intensive and may not be optimal for repeated or rapid targeted testing.^3^^,^^4^^,^^5^ In addition, the multiplex STA-HiDy-CE system offered a substantially shorter turnaround time compared with targeted amplicon sequencing (4.5 h vs. 24 h; Table S8), supporting its potential utility for rapid clinical testing. These differences indicate that the multiplex STA-HiDy-CE system can provide a substantially faster analytical workflow. In addition, its ability to quantitatively detect multiple low-frequency variants in clinical specimens supports its potential use as a targeted molecular analysis platform, particularly for small, disease-focused mutation panels or as a prescreening tool to identify specimens that may warrant more comprehensive genomic analysis.

Importantly, although targeted amplicon sequencing and digital PCR tend to show higher sensitivity in the very low-frequency range below 1% VAF, detection at approximately 1% VAF remains clinically meaningful. For tissue specimens, the clinical relevance of this assay lies in complementing pathological diagnosis and prescreening samples for CGP. Small lesions with VAFs of a few percent may be missed or remain inconclusive in pathological evaluation. In our previous study, HiDy-CE detected mutations even in cases in which pathological diagnosis was inconclusive.^10^ Thus, detection at around 1% VAF may be sufficient for this purpose. In addition, because CGP is costly and generally requires samples with a relatively high tumor cell fraction, prescreening specimens suitable for CGP would be beneficial. In this context, our system may serve as a simple and cost-effective screening tool. For liquid specimens, the assay may also help reduce missed detections in screening settings, because mutations with VAFs greater than 1% can be observed in plasma from patients with advanced cancer.

Several limitations of this study should be acknowledged. First, the number of target loci evaluated was limited, and additional validation will be required as multiplexing capacity is expanded. Second, false-positive calls were observed in both FFPE and liquid specimens. Mutation-calling thresholds were determined using WT synthetic ssDNA, because assembling a large set of variant-negative clinical samples may not be practical in an individual laboratory setting. Although discordant mutation calls were observed when compared with targeted amplicon sequencing and digital PCR, the rates of discordant calls were low (1.41% in FFPE specimens and 2.26% in liquid specimens), and the thresholding strategy yielded acceptable performance in clinical specimens. Further refinement of signal-processing algorithms to better distinguish true variant signals from background noise may reduce false-positive calls. Third, false-negative results were observed in liquid specimens. Although all variants with VAFs of 1% or greater were detected, detection of variants with VAFs below 1% was variable. Because low-level mutations in liquid specimens may still be clinically relevant,^21^ further optimization of variant-specific thresholds, signal-processing methods, or paired tumor-normal comparisons may improve performance for variants at very low-allele frequencies.

In conclusion, this study demonstrates that integrating multiplex STA chemistry with HiDy-CE enables simultaneous and quantitative detection of multiple low-frequency variants across clinically relevant loci within a single electrophoretic run. This approach provides a scalable framework for targeted mutation analysis using limited clinical material, offering a cost-effective alternative to CGP for disease-focused gene panels. Further refinement of assay design and signal-processing algorithm holds promise for expanding multiplexing capacity and broadening the clinical utility of this platform.

## Materials and methods

### Patient samples

FFPE tissue samples were obtained from 38 patients with resectable pancreatic disease who were treated at Asahikawa Medical University between 2018 and 2021. Liquid specimens, including pancreatic juice and duodenal fluid, were collected from 10 patients and analyzed in this study. Tumor samples harboring mutations in *KRAS* codons 12 and 13 and/or *GNAS* codon 201 were selected for analysis.

gDNA was extracted from unstained FFPE sections (10 μm thick; 1–5 sections per case) using the GeneRead DNA FFPE kit (QIAGEN, Hilden, Germany).^22^ Duodenal fluid samples were aspirated endoscopically using an MMI tracheal suction kit (Muranaka Medical Instruments, Tokyo, Japan) and mixed with Tris-EDTA (TE) buffer (100 mmol/L Tris-HCl, 50 mmol/L EDTA, pH 8.0) to a final volume of 3–5 mL. Pancreatic juice samples were collected via an endoscopic retrograde cholangiopancreatography (ERCP) catheter and frozen within 10 min after collection. gDNA from liquid samples was extracted using the QIAamp MinElute ccfDNA Midi Kit (QIAGEN). Purified gDNA was quantified using a Qubit dsDNA HS Assay Kit on a Qubit 4 Fluorometer (Thermo Fisher Scientific, Waltham, MA, USA), according to the manufacturer’s instructions.

All patient-derived specimens were collected after written informed consent was obtained from the participants. The study was approved by the Research Ethics of Asahikawa Medical University Committee (approval #21055 and #21138) and conducted in accordance with the Declaration of Helsinki.

### Control sample preparation

Synthesized single-stranded oligonucleotides (ssDNA) harboring *KRAS* and *GNAS* mutations listed in Table S1 were purchased from Integrated DNA Technologies (Coralville, IA, USA) and used to generate a calibration curve in a dilution series experiment. Variant and corresponding WT ssDNA were mixed to produce final VAFs ranging from 0.1% to 8% for all variants included in the analysis.

### Multiplex STA

PCR amplification and STA reactions were performed using the Mastercycler ep Gradient S Thermal Cycler (Eppendorf, Hamburg, Germany). A reagent system modified from the *KRAS* Mutation Detection Kit (TrimGen, MD, USA), as illustrated in Figure 1, was used for multiplex mutation analysis. Primer sequences for PCR and STA reactions are listed in Table S2.

gDNA was diluted with Ambion nuclease-free water (Thermo Fisher Scientific) to a final concentration of 5 ng/μL. Detailed descriptions of the PCR reaction, post-PCR cleanup and STA protocols are provided in Table S9.

STA products were purified by centrifugation using TF-50 filter tips at 1,000× g for 2.5 min. Finally, 3 μL from each STA reaction solution were combined, mixed with 12.5 μL of Hi-Di Formamide (Thermo Fisher Scientific) and 0.5 μL of GeneScan 120 LIZ dye Size Standard (Thermo Fisher Scientific), and denatured at 94°C for 3 min prior to CE analysis.

### HiDy-CE

HiDy-CE shared the same hardware configuration as the compact CE sequencer DS3000 (Hitachi High-Tech, Tokyo, Japan), which is equipped with four 36-cm-long capillaries and a charge-coupled device (CCD) image sensor for fluorescence detection. In contrast to the standard DS3000 configuration, HiDy-CE expands the dynamic range of fluorescence detection by modifying the operating mode of the CCD image sensor, as previously described.^10^

Signal acquired from the 240 hardware binning regions per capillary were combined in groups of 12 and subsequently deconvoluted into four dye-specific signals to mitigate spectral overlap among the four fluorescent dyes.^10^

### Electrophoresis and data analysis

Electrophoresis was performed using the HiDy-CE system. Electrophoresis conditions were as follows: injection voltage, 1.6 kV; injection time, 9 s; run voltage, 12.5 kV; run time, 1,280 s; and oven temperature, 55°C. POP-6 polymer (Thermo Fisher Scientific) was used as the separating matrix.

Raw fluorescence intensities were normalized so that the peak height of the WT allele was scaled to 1 for each of the three loci (*KRAS* codons 12 and 13, and *GNAS* codon 201). The same normalization was applied to WT synthesized ssDNA that was electrophoresed simultaneously. Frames were shifted so that the peak top frame of the WT allele aligned across samples at each locus. After alignment, the relative intensities of each frame in the test sample were baseline-corrected by subtracting the corresponding frame relative values from the WT synthesized ssDNA standard. Variant alleles were identified by defining allele-specific detection windows derived from calibration curve generated using synthesized mutant ssDNA standards. For each allele, the detection window was defined as the mean frame size ±3 standard deviations (SDs) (Table S1; Figure S2). The maximum relative intensity within each predicted window was used as representative peak height for the corresponding allele.

The total turnaround time from sample preparation to data analysis was approximately 4.5 h for the multiplex STA-HiDy-CE assay, compared with approximately 24 h for targeted amplicon sequencing (Table S8).

### Correction coefficients for peak height bias

To correct systematic differences in peak height among variants, variant-specific correction coefficients were calculated for each mutant allele. The coefficient *k*_*MT*_ was defined as the relative peak height of a mutant-type (MT) allele compared with the WT allele under equal allele proportion. Let *x* denote the input VAF, and let *S*_*MT*_ denote the relative height of the MT peak normalized to the WT peak. Then, *k*_*MT*_ was calculated as:

(Equation 1) $$k_{MT}=\frac{S_{MT}}{\left(1-6x\right)/x}\text{.}$$

For *KRAS*, in which 6 variants were mixed simultaneously, Equation 1 was applied. For *GNAS*, in which 3 variants were mixed simultaneously, *k*_*MT*_ was calculated as:

(Equation 2) $$k_{MT}=\frac{S_{MT}}{\left(1-3x\right)/x}\text{.}$$

In the dilution series experiments, the input VAF (*x*) was predefined, and *S*_*MT*_ were obtained from electropherograms. Correction coefficients for each variant were estimated using the least-squares method (Table S4).

### VAFs calculation

Calculated VAF were determined using the variant-specific correction coefficients. For *KRAS* codon 12 variants, the calculated VAF for a given variant *X* was defined as:

(Equation 3) $${Calculated\;VAF}_{G12X}=\frac{S_{G12X}/k_{G12X}}{\left(S_{G12V}/k_{G12V}\right)+\left(S_{G12D}/k_{G12D}\right)+\left(S_{G12R}/k_{G12R}\right)+\left(S_{G12A}/k_{G12A}\right)+\left(S_{G12C}/k_{G12C}\right)+\left(S_{G12S}/k_{G12S}\right)+1}\text{.}$$

The same calculation was applied to *KRAS* codon 13 variants.

For *GNAS* variants, the calculated VAF for a given variant *X* was defined as:

(Equation 4) $${Calculated\;VAF}_{R201X}=\frac{S_{R201X}/k_{R201X}}{\left(S_{R201H}/k_{R201H}\right)+\left(S_{R201C}/k_{R201C}\right)+\left(S_{R201S}/k_{R201S}\right)+1}\text{.}$$

### Noise cancellation

Noise patterns were acquired using samples containing only *KRAS* G12V or *GNAS* R201C. A cancellation matrix was constructed to eliminate the prominent noise signals, particularly the G12D noise induced by the G12V and the R201S noise induced by the R201C (Figure S1; Table S3).

Noise was removed by matrix-based computation. Let matrix *A* represent the relationship between the true proportions of the 15 variants in the matrix-construction samples and the VAFs of the 15 variants observed in the corresponding electropherograms of the matrix-construction samples. Let matrix *X* represent the true VAFs of the 15 variants contained in the sample under analysis, and let matrix *Y* represent the observed VAFs of the 15 variants obtained from the electropherogram of those samples. Then, *X* was estimated from *Y* using the inverse matrix of *A* as follows:

(Equation 5) $$X=A^{-1}\times Y\text{.}$$

### Mutation-calling threshold

Mutation-calling threshold was determined from WT-synthesized ssDNA samples. Across 10 replicate measurements, the maximum VAF among 15 variant alleles was 0.42% (Figure S3). To reduce false-positives, the threshold for calling as mutation was set at 0.5%. For FFPE samples, each sample was measured twice, and an allele was considered as mutation only when it exceeded the threshold in both measurements. For liquid samples, each sample was measured once, and allele exceeding the threshold was considered as mutation. Sensitivity and specificity were calculated using targeted amplicon sequencing results as the reference for FFPE tissue specimens and digital PCR results as the reference for liquid specimens, because digital PCR provides superior sensitivity for low-VAF variants in liquid specimens.

### Quantitative analysis using control sample

Each VAF level was measured in triplicate on 2 separate days, resulting in 6 replicates per variant at each VAF level. In total, 450 measurements were obtained across all variants and VAF levels. For each VAF level, within-run CVs and total CVs were calculated to evaluate precision. Sensitivity and accuracy were also calculated for each VAF level. Accuracy was defined as the relative difference between the mean calculated VAF and the nominal input VAF, as follows:

(Equation 6) $$Accuracy\;deviation\;\left(\%\right)=\frac{\left|calculated\;VAF-nominal\;input\;VAF\right|}{nominal\;input\;VAF}\times 100\text{.}$$

The LOQ was defined as the lowest input VAF at which both the accuracy deviation and CV were ≤20%.

### Targeted amplicon sequencing and digital PCR

Mutation profiling of FFPE tumor and matched normal tissues from surgically resected specimens, as well as liquid samples, were characterized using custom targeted amplicon sequencing panel targeting eight genes associated with PDA,^21^ and for liquid samples additionally with single-plex digital PCR using previously reported procedures.^23^ For mutation profiling, 10 ng of gDNA for tissue specimens and 40 ng of gDNA for liquid specimens were used.

## Data and code availability

The data supporting the findings of this study are available from the corresponding author upon reasonable request.

## Acknowledgments

We thank members in Division of Gastroenterology, Asahikawa Medical University for their helpful discussions during the project work. This work was conducted by research funding from 10.13039/100031117Hitachi High-Tech Corporation, Hitachi Inc, 10.13039/501100001691KAKENHI grant nos. 23K06759, 24K02431, 22KK0125, Princess Takamatsu Cancer Research fund, The Naito Grant for the Advancement of Natural Science, and Project Mirai Cancer Research grants.

## Author contributions

Conceptualization, N.S. and H. Kato; data curation, Y.H. and Y. Ono; formal analysis, Y.H. and Y. Ono; funding acquisition, Y. Ono and Y.M.; investigation, Y.H., N.T., C. Maeda, M.M., C. Matsumoto, M.S., and S.C.; methodology, Y.H.; project administration, N.S. and Y.M.; resources; N.T., Y.S., K.K., Y. Ohtaki, T.O., H. Kawabata, and M.T.; supervision, N.S., Y. Ono, M.Y., and Y.M.; validation, C. Maeda, M.M., C. Matsumoto, M.S., and S.C.; visualization, Y.H.; writing – original draft, Y.H. and N.T.; writing – review and editing, all authors.

## Declaration of interests

Y.H., N.S., H. Kato, and M.Y. are employees of Hitachi High-Tech Corporation.

Y. Ono has received research funding from Hitachi High-Tech Corporation and KAKENHI grant no. 23K06759.

K. Takahashi and Y.S. have received research funding from Hitachi High-Tech Corporation.

Y.M. has received research funding from Hitachi High-Tech Corporation and Hitachi, Ltd., as well as support from KAKENHI grant nos. 24K02431 and 22KK0125, Princess Takamatsu Cancer Research Fund, The Naito Grant for the Advancement of Natural Science, and Project Mirai Cancer Research Grants.

Y.H., Y. Ono, K. Takahashi, H. Kat., M.Y., and Y.M. are co-inventors on a pending patent application (PCT/JP2025/018802) related to the technology described in this manuscript.

Y.H. and N.S. are co-inventors of pending patent application (PCT/JP2026/008144) related to the technology described in this manuscript.

## Supplemental information

Document S1. Figures S1–S3 and Tables S1–S8

Table S5. Results of multiplex STA-HiDy-CE system’s mutation calling for each sample and each variant using tissue specimens, see standalone Table S5

Document S2. Article plus supplemental information
