# Supplementary material for: Simultaneous quantitative detection of multiple low-frequency variants by high-dynamic-range capillary electrophoresis
Source: Mol Ther Nucleic Acids. 2026 Jul 30;37(3):103042. doi: 10.1016/j.omtn.2026.103042 (PMC13542593; doi:10.1016/j.omtn.2026.103042)
Supplement: Supplementary file 1 [file mmc1.pdf]

OMTN, Volume *37*

Supplemental information

Simultaneous quantitative detection of multiple low-frequency variants by high-dynamic-range capillary electrophoresis

Yoshihiko Hagiwara, Nobue Tamamura, Noriyuki Sumida, Yusuke Ono, Kenji Takahashi, Yanpeng Sun, Kazuya Koyama, Yu Ohtaki, Tetsuhiro Okada, Hidemasa Kawabata, Chiho Maeda, Miyuki Mori, Chiemi Matsumoto, Mayumi Suzuki, Shin-ichi Chiba, Mishie Tanino, Kenzui Taniue, Hirokazu Kato, Motohiro Yamazaki, and Yusuke Mizukami

Supplemental Figures

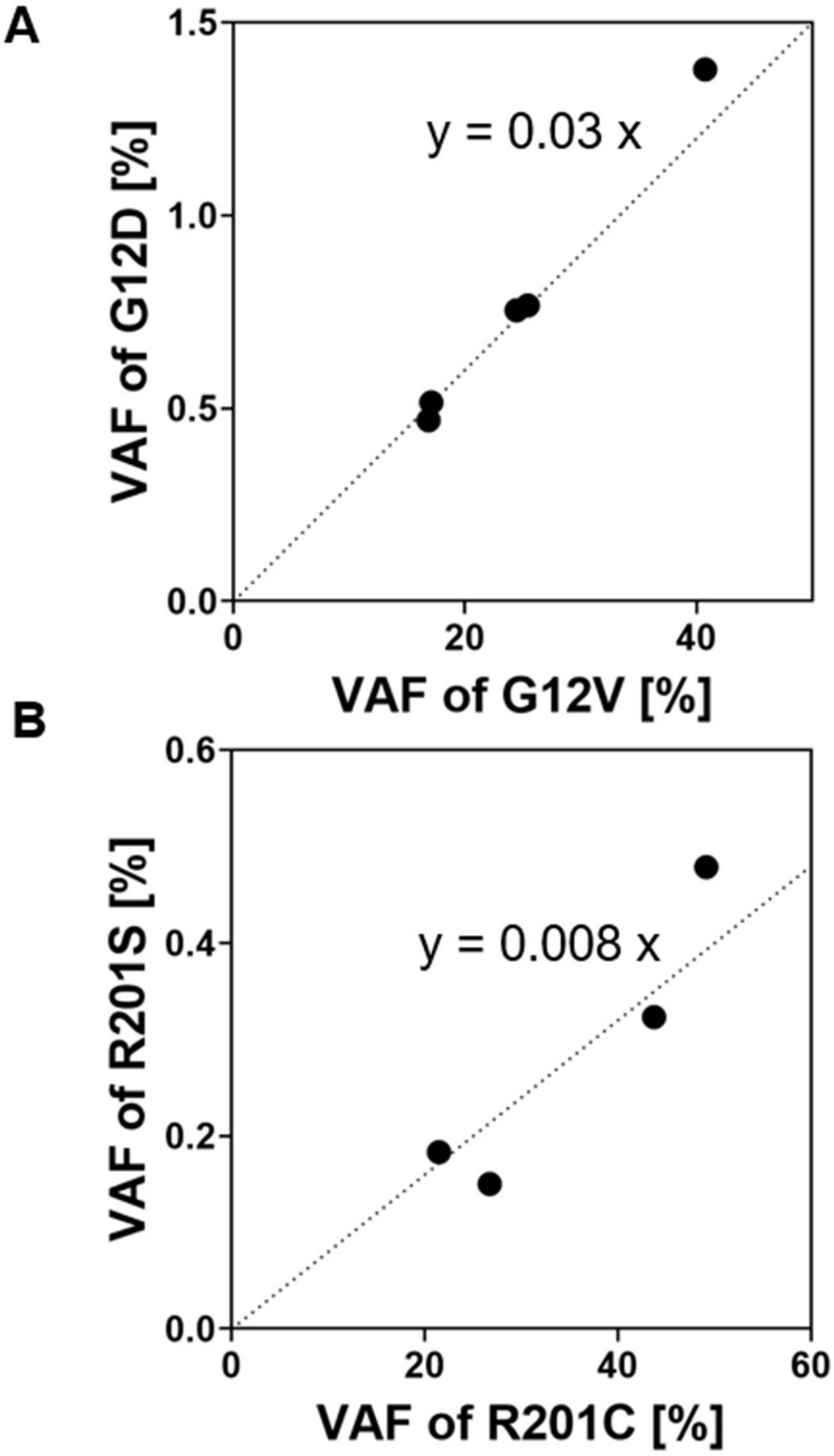

Figure S1. Relation between the VAF of noise-generating variant and the resulting noise signal. (A) Dot plots showing the relationship between the variant allele frequencies (VAFs) of *KRAS* G12V and G12D in samples containing only the G12V mutation. (B) Dot plots showing the relationship between the VAFs of *GNAS* R201C and R201S in samples containing only the R201C mutation.

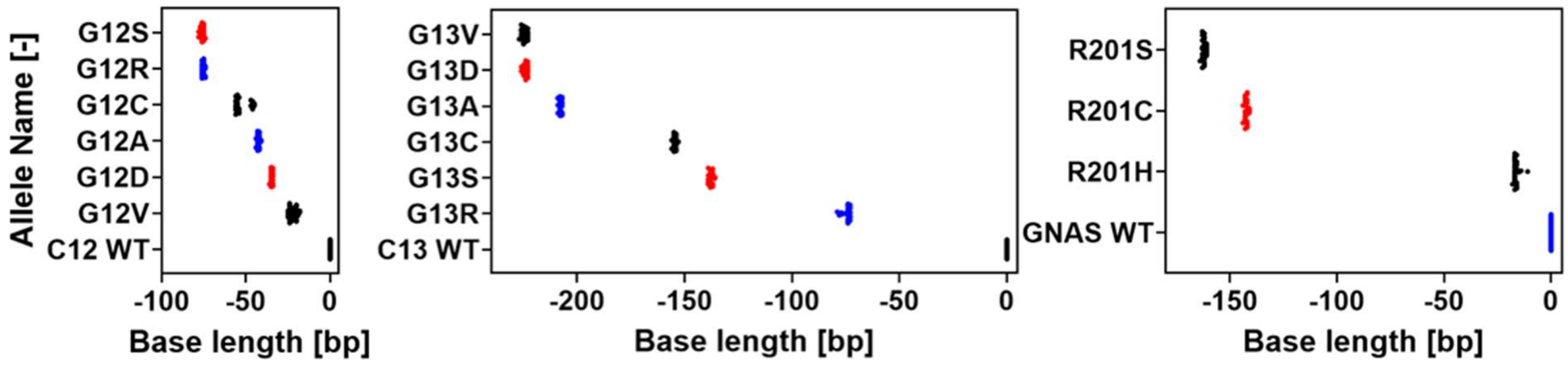

Figure S2. Distribution of peak position for each allele on electropherograms.

Dot plots illustrate the positions of each allele. The plots represent results from 30 replicate measurements of samples containing variant allele frequency from 0.1% to 8%. For each allele, the peak detection range was set to the mean ± 3 standard deviation of its position.

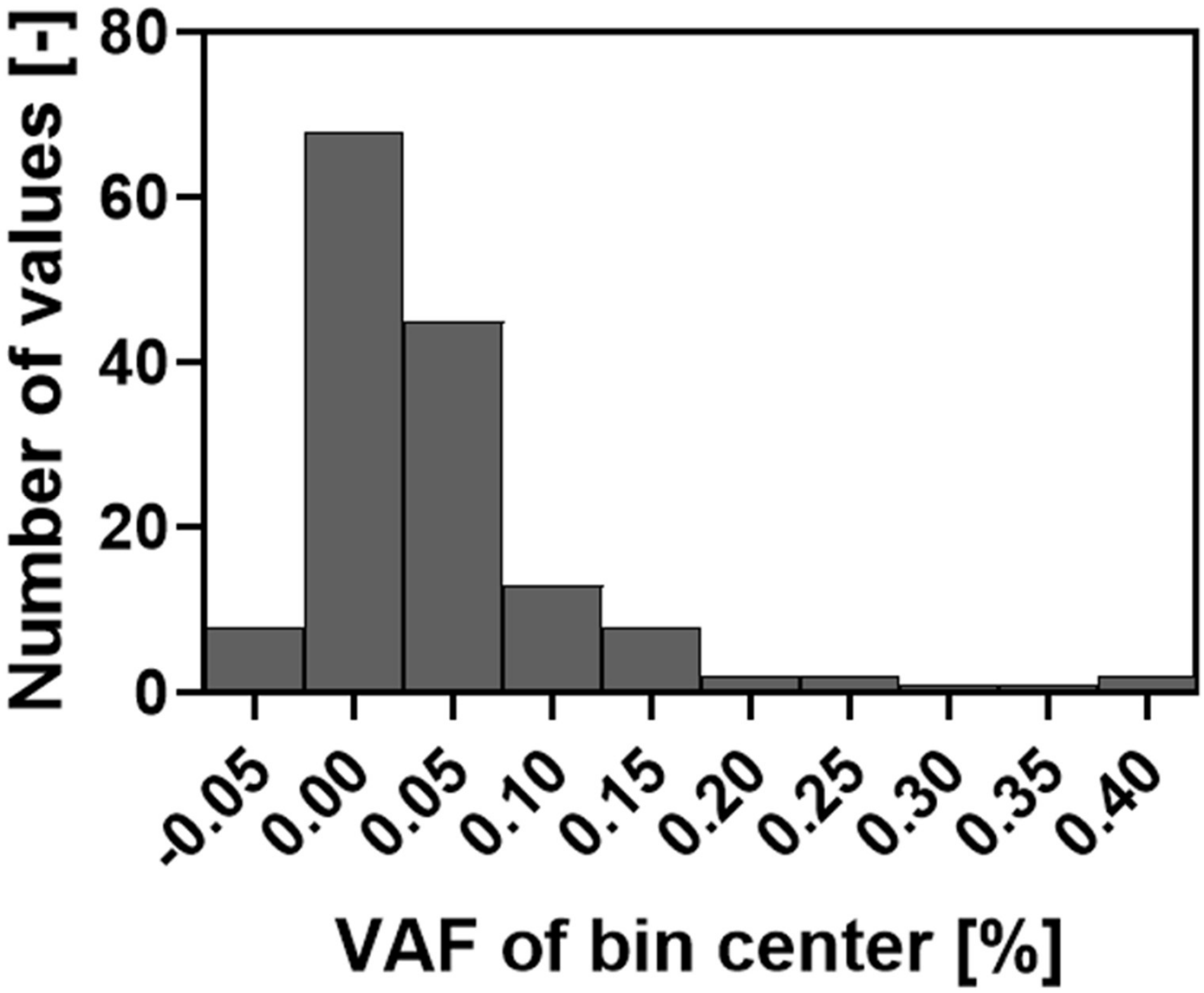

Figure S3. Distribution of calculated VAFs in wild-type control sample.

Histogram showing the calculated VAFs for all variants when using wild-type synthetic single-stranded oligonucleotides. The data represent 10 replicate measurements.

Supplemental Tables

Table S1. All Detectable Alleles and Sequences of Single-stranded Oligonucleotides.

Bold font indicates the mutation site.

Target	Sequence for DNA Fragment (5'-sequence-3')	Fluorescence Color	Start Position [relative frame]	End Position [relative frame]
KRAS Wild-type (Codon12)	GTCACATTTTCATTATTTTTATTATAAGGCCTGCTGAAAATGACTGAATATAAACTTGTGG TAGTTGGAGCTGGTGGCGTAGGCAAGAGTGCCTTGACGATAC	Yellow	-	-
KRAS G12S	GTCACATTTTCATTATTTTTATTATAAGGCCTGCTGAAAATGACTGAATATAAACTTGTGG TAGTTGGAGCT**A**GTGGCGTAGGCAAGAGTGCCTTGACGATAC	Red	-78	-72
KRAS G12R	GTCACATTTTCATTATTTTTATTATAAGGCCTGCTGAAAATGACTGAATATAAACTTGTGG TAGTTGGAGCT**C**GTGGCGTAGGCAAGAGTGCCTTGACGATAC	Blue	-78	-72
KRAS G12C	GTCACATTTTCATTATTTTTATTATAAGGCCTGCTGAAAATGACTGAATATAAACTTGTGG TAGTTGGAGCT**T**GTGGCGTAGGCAAGAGTGCCTTGACGATAC	Yellow	-58	-52
KRAS G12D	GTCACATTTTCATTATTTTTATTATAAGGCCTGCTGAAAATGACTGAATATAAACTTGTGG TAGTTGGAGCTG**A**TGGCGTAGGCAAGAGTGCCTTGACGATAC	Red	-38	-31
KRAS G12A	GTCACATTTTCATTATTTTTATTATAAGGCCTGCTGAAAATGACTGAATATAAACTTGTGG TAGTTGGAGCTG**C**TGGCGTAGGCAAGAGTGCCTTGACGATAC	Blue	-45	-40
KRAS G12V	GTCACATTTTCATTATTTTTATTATAAGGCCTGCTGAAAATGACTGAATATAAACTTGTGG TAGTTGGAGCTG**T**TGGCGTAGGCAAGAGTGCCTTGACGATAC	Yellow	-28	-20

KRAS Wild-type (Codon13)	Same as KRAS wild-type (Codon12)	Yellow	-	-
KRAS G13S	GTCACATTTTCATTATTTTTATTATAAGGCCTGCTGAAAATGACTGAATATAAACTTGTGG TAGTTGGAGCTGGT**A**GCGTAGGCAAGAGTGCCTTGACGATAC	Red	-141	-134
KRAS G13R	GTCACATTTTCATTATTTTTATTATAAGGCCTGCTGAAAATGACTGAATATAAACTTGTGG TAGTTGGAGCTGGT**C**GCGTAGGCAAGAGTGCCTTGACGATAC	Blue	-75	-71
KRAS G13C	GTCACATTTTCATTATTTTTATTATAAGGCCTGCTGAAAATGACTGAATATAAACTTGTGG TAGTTGGAGCTGGT**T**GCGTAGGCAAGAGTGCCTTGACGATAC	Yellow	-157	-152
KRAS G13D	GTCACATTTTCATTATTTTTATTATAAGGCCTGCTGAAAATGACTGAATATAAACTTGTGG TAGTTGGAGCTGGTG**A**CGTAGGCAAGAGTGCCTTGACGATAC	Red	-227	-221
KRAS G13A	GTCACATTTTCATTATTTTTATTATAAGGCCTGCTGAAAATGACTGAATATAAACTTGTGG TAGTTGGAGCTGGTG**C**CGTAGGCAAGAGTGCCTTGACGATAC	Blue	-210	-205
KRAS G13V	GTCACATTTTCATTATTTTTATTATAAGGCCTGCTGAAAATGACTGAATATAAACTTGTGG TAGTTGGAGCTGGTG**T**CGTAGGCAAGAGTGCCTTGACGATAC	Yellow	-229	-220
GNAS Wild-type	TCCATTGACCTCAATTTTGTTTCAGGACCTGCTTCGCTGCCGTGTCCTGACTTCTGGA ATCTTTGAGACCAAGTTCCAGGTGGACAAAGTCAACTTCCAGTAAGCCAACT	Yellow	-	-
GNAS R201S	TCCATTGACCTCAATTTTGTTTCAGGACCTGCTTCGCTGC**A**GTGTCCTGACTTCTGGA ATCTTTGAGACCAAGTTCCAGGTGGACAAAGTCAACTTCCAGTAAGCCAACT	Red	-141	-134
GNAS R201C	TCCATTGACCTCAATTTTGTTTCAGGACCTGCTTCGCTGC**T**GTGTCCTGACTTCTGGA ATCTTTGAGACCAAGTTCCAGGTGGACAAAGTCAACTTCCAGTAAGCCAACT	Blue	-75	-71
GNAS R201H	TCCATTGACCTCAATTTTGTTTCAGGACCTGCTTCGCTGCC**A**TGTCCTGACTTCTGGA ATCTTTGAGACCAAGTTCCAGGTGGACAAAGTCAACTTCCAGTAAGCCAACT	Yellow	-157	-152

Table S2. Sequences and Concentration of Primers for PCR and STA.

Reaction	Primers	Sequence of DNA Fragment (5'-sequence-3')	Final Concentration (μM)
PCR	*KRAS* Forward PCR	GTCACATTTTCATTATTTTTATTATAAGG	0.2
PCR	*KRAS* Reverse PCR	GTATCGTCAAGGCACTCTTGCC	0.2
PCR	*GNAS* Forward PCR	TCCATTGACCTCAATTTTGTTTC	0.2
PCR	*GNAS* Reverse PCR	AGTTGGCTTACTGGAAGTTG	0.2
STA	*KRAS* C12 STA	CTCTCTCTCTCTCTCTCTCTCTCTCTAACTTGTGGTAGTTGGAGCT	0.9
STA	*KRAS* C13 STA	TCTCTCTCTCTCTCTCTCTCTCTCTCTCTCTCTCTTCAAGGCACTCTTGCCTACG	0.45
STA	*GNAS* C201 STA	TTTTTTTTTTTTTTTTTTTTTTTTTTTTTTTTTTTTTTTTTTTTTTTTTTTTTAGGACC TGCTTCGCTGC	0.45

Table S3. Noise Cancellation Matrix.

* The proportion of the 15 variants present in matrix-construction samples.

Gene	Variant	VAFs of the 15 variants observed in the electropherograms of matrix-construction samples*														
		KRAS Codon 12						*KRAS* Codon 13						*GNAS* Codon 201		
		G12V	G12D	G12A	G12C	G12R	G12S	G13R	G13S	G13C	G13A	G13D	G13V	R201H	R201C	R201S
KRAS	G12V	1	0.03	0	0	0	0	0	0	0	0	0	0	0	0	0
Codon 12	G12D	0	1	0	0	0	0	0	0	0	0	0	0	0	0	0
	G12A	0	0	1	0	0	0	0	0	0	0	0	0	0	0	0
	G12C	0	0	0	1	0	0	0	0	0	0	0	0	0	0	0
	G12R	0	0	0	0	1	0	0	0	0	0	0	0	0	0	0
	G12S	0	0	0	0	0	1	0	0	0	0	0	0	0	0	0
KRAS	G13R	0	0	0	0	0	0	1	0	0	0	0	0	0	0	0
Codon 13	G13S	0	0	0	0	0	0	0	1	0	0	0	0	0	0	0
	G13C	0	0	0	0	0	0	0	0	1	0	0	0	0	0	0
	G13A	0	0	0	0	0	0	0	0	0	1	0	0	0	0	0
	G13D	0	0	0	0	0	0	0	0	0	0	1	0	0	0	0
	G13V	0	0	0	0	0	0	0	0	0	0	0	1	0	0	0
GNAS	R201H	0	0	0	0	0	0	0	0	0	0	0	0	1	0	0
Codon 201	R201C	0	0	0	0	0	0	0	0	0	0	0	0	0	1	0.008
	R201S	0	0	0	0	0	0	0	0	0	0	0	0	0	0	1

Table S4. Variant-specific Correction Coefficient Applied to Each Variant for Calculating Variant Allele Frequency.

KRAS Codon 12						*KRAS* Codon 13						*GNAS* Codon201		
G12S	G12R	G12C	G12D	G12A	G12V	G13S	G13R	G13C	G13D	G13A	G13V	R201S	R201C	R201H
1.97	4.13	1.06	1.37	7.73	0.853	0.652	7.40	4.03	1.19	4.24	0.648	0.668	0.845	1.47

Table S5. Results of Multiplex STA-HiDy-CE System's Mutation Calling for Each Sample and Each Variant Using Tissue Specimens.

See Standalone Table S5.

Table S6. Sensitivity and Specificity of Multiplex STA-HiDy-CE System Using Clinical Samples.

	FFPE specimens	Liquid specimens
Count of True Positives (allele)	34	13
Count of False Positives (allele)	8	3
Count of True Negative (allele)	558	131
Count of False Negative (allele)	0	3
Sensitivity (%)	100	76.47
Specificity (%)	98.59	97.74

Table S7. Results of Multiplex STA-HiDy-CE System's Mutation Calling for Each Sample and Each Variant Using Liquid Specimens. Bold font indicates true positive, underline indicates false positive, and Italic font indicates false negative. *KRAS* G12A, G12C, G12S, G13R, G13S, G13C, G13A, G13D, G13V, and *GNAS* R201S were not analyzed on digital PCR.

Sample	Variant														
	KRAS Codon 12						*KRAS* Codon 13						*GNAS* Codon 201		
	G12V	G12D	G12A	G12C	G12R	G12S	G13R	G13S	G13C	G13A	G13D	G13V	R201H	R201C	R201S
41	**1.42%**	-0.10%	0.03%	0.02%	-0.05%	0.17%	0.00%	-0.24%	-0.11%	0.03%	0.04%	0.11%	-0.44%	0.06%	0.05%
42	**30.00%**	**0.80%**	0.02%	0.31%	*0.22%*	<u>0.72%</u>	-0.01%	-0.31%	-0.05%	0.01%	-0.02%	0.22%	0.37%	0.18%	0.18%
43	0.11%	-0.08%	0.03%	0.01%	-0.06%	0.15%	0.01%	-0.14%	-0.09%	0.01%	0.06%	0.19%	-0.48%	-0.01%	0.12%
44	0.18%	*-0.03%*	-0.01%	0.00%	-0.07%	<u>0.58%</u>	-0.01%	-0.24%	-0.04%	0.01%	0.07%	0.14%	-0.02%	0.08%	0.09%
45	0.10%	**1.83%**	0.03%	0.04%	0.06%	0.25%	0.02%	-0.38%	-0.07%	0.05%	0.02%	0.33%	-0.51%	0.02%	0.02%
46	0.18%	*-0.04%*	0.01%	-0.01%	-0.04%	0.19%	-0.01%	-0.28%	-0.06%	0.01%	-0.01%	0.13%	-0.05%	0.07%	0.06%
47	**2.69%**	-0.08%	0.01%	0.03%	-0.05%	0.15%	0.00%	-0.33%	-0.09%	0.01%	0.00%	0.17%	-0.10%	**3.71%**	0.08%
48	**2.53%**	-0.05%	-0.04%	0.04%	-0.06%	0.24%	0.00%	-0.20%	-0.06%	0.00%	0.08%	0.13%	**0.68%**	**3.40%**	0.10%
49	**9.65%**	**1.57%**	0.01%	0.03%	-0.07%	<u>0.52%</u>	-0.01%	-0.47%	-0.05%	0.02%	0.09%	0.20%	**24.51%**	*0.30%*	0.11%
50	**6.15%**	-0.10%	-0.01%	0.00%	-0.06%	0.42%	0.00%	-0.20%	-0.06%	0.00%	0.03%	0.10%	-0.08%	0.02%	0.04%

Table S8. Comparison of Turnaround Time between Multiplex STA-HiDy-CE System and Targeted Amplicon Sequencing.

Step	Turnround Time (h)	
	Multiplex STA-HiDy-CE System	Targeted Amplicon Sequencing
Sample preparation	3.5	6
Instrument run time including preparation	0.5	16
Data analysis	0.5	2
Total	4.5	24

Table S9. Experimental Condition for Multiplex STA-HiDy-CE System.

*: For samples with DNA template concentrations below 5 ng/μL, the input volume of DNA was increased so that a total of 10 ng of DNA could be used for the PCR reaction.

PCR mixture	Reagents	Volume (μL)	
	Master mix	18	
	KRAS Forward PCR primer	0.25	
	KRAS Reverse PCR primer	0.25	
	GNAS Forward PCR primer	0.25	
	GNAS Reverse PCR primer	0.25	
	DNA template	2*	
	Total	21	
PCR condition	Temperature (℃)	Duration	Cycle
	94	5 min	X 1
	94	30 s	
	52	30 s	X 39
	72	30 s	
	72	5 min	X 1
	4	∞	
Post-PCR clean-up mixture	Reagents	Volume (μL)	
	Clean-up Enzymes	11	
	PCR products	4	
	Total	15	
Post-PCR condition	Temperature (℃)	Duration	
	37	25 min	
	95	5 min	
	4	∞	
STA mixture tube #1	Reagents	Volume (μL)	
	KRAS EM-12	11	
	KRAS C12 STA primer	2	
	Post-PCR cleaned products	2	
	Total	15	

STA mixture tube #2	Reagents	Volume (μL)	
	KRAS EM-13	11	
	KRAS C13 STA primer	1	
	GNAS C201 STA primer	1	
	Post-PCR cleaned products	2	
	Total	15	
STA condition	Temperature (℃)	Duration	Cycle
	94	4 min	X 1
	94	20 s	
	60	20 s	X 20
	70	20 s	
	4	∞	
